# Increased PD-1^+^Foxp3^+^ γδ T cells associate with poor overall survival for patients with acute myeloid leukemia

**DOI:** 10.3389/fonc.2022.1007565

**Published:** 2022-12-15

**Authors:** Jiamian Zheng, Dan Qiu, Xuan Jiang, Yun Zhao, Haotian Zhao, Xiaofang Wu, Jie Chen, Jing Lai, Wenbin Zhang, Xutong Li, Yangqiu Li, Xiuli Wu, Zhenyi Jin

**Affiliations:** ^1^ Key Laboratory for Regenerative Medicine of Ministry of Education, Institute of Hematology, School of Medicine, Jinan University, Guangzhou, China; ^2^ Department of Traditional Chinese Medicine, Heyuan People’s Hospital, Heyuan, China; ^3^ Department of Hematology, First Affiliated Hospital, Jinan University, Guangzhou, China; ^4^ Department of Oncology, First Affiliated Hospital, Jinan University, Guangzhou, China; ^5^ Department of Pathology, School of Medicine, Jinan University, Guangzhou, China

**Keywords:** acute myeloid leukemia, γδ T cells, PD-1, Foxp3, outcome, overall survival

## Abstract

**Problems:**

γδ T cells are essential for anti-leukemia function in immunotherapy, however, γδ T cells have different functional subsets, including regulatory cell subsets expressing the Foxp3. Whether they are correlated with immune-checkpoint mediated T cell immune dysfunction remains unknown in patients with acute myeloid leukemia (AML).

**Methods:**

In this study, we used RNA-seq data from 167 patients in TCGA dataset to analyze the correlation between *PD-1* and *FOXP3* genes and these two genes’ association with the prognosis of AML patients. The expression proportion of Foxp3^+^/PD-1^+^ cells in γδ T cells and two subgroups Vδ1 and Vδ2 T cells were performed by flow cytometry. The expression level of *FOXP3* and *PD-1* genes in γδ T cells were sorted from peripheral blood by MACS magnetic cell sorting technique were analyzed by quantitative real-time PCR.

**Results:**

We found that *PD-1 *gene was positively correlated with *FOXP3* gene and highly co-expressed *PD-1* and *FOXP3* genes were associated with poor overall survival (OS) from TCGA database. Then, we detected a skewed distribution of γδ T cells with increased Vδ1 and decreased Vδ2 T cell subsets in AML. Moreover, significantly higher percentages of PD-1^+^ γδ, Foxp3^+^ γδ, and PD-1^+^Foxp3^+^ γδ T cells were detected in *de novo* AML patients compared with healthy individuals. More importantly, AML patients containing higher PD-1^+^Foxp3^+^ γδ T cells had lower OS, which might be a potential therapeutic target for leukemia immunotherapy.

**Conclusion:**

A significant increase in the PD-1^+^Foxp3^+^ γδ T cell subset in AML was associated with poor clinical outcome, which provides predictive value for the study of AML patients.

## Introduction

Acute myeloid leukemia (AML) is an aggressive hematological malignancy characterized by the accumulation of immature myeloid precursors, ultimately resulting in inhibition of normal hematopoiesis. Although understanding of the classification, molecular mechanism, pathobiology, and genomic landscape of AML has advanced in recent years, the treatment standard has remained upfront induction chemotherapy followed by consolidation chemotherapy or hematopoietic stem cell transplantation (HSCT) (1–4). In addition, there is no regimen that can effectively protect against poor outcome with relapse/refractory disease and the achievement of sustained complete remission (CR) for AML patients (5, 6). According to the clinical trials, the median survival months of AML patients with relapse or refractory were less than 10 months (3, 7–11). In general, allogeneic HSCT achieves survival in 20%-35% of relapse/refractory AML patients at 4 years (12, 13). Thus, relapse after allogeneic HSCT is a common problem and occurs in 25%-55% of AML patients. It has been shown that leukemia cells induce the expression of immune checkpoint (IC) genes and the immune escape of leukemia cells is a key cause of relapse and refractory. Recently, blockades of immune-checkpoint inhibitors (ICIs), such as programmed death receptor (PD-1) and its ligand (PD-L1), T-cell immunoreceptor with immunoglobulin and immunoreceptor tyrosine-based inhibitory motif (ITIM) domain (TIGIT), and T cell immunoglobulin and mucin domain (Tim-3), have been proven to be successful in the treatment of solid tumors (14, 15). In contrast, the clinical effectiveness of such immune therapies appears to be relatively different for AML subtypes and clinical trials with different prognosis (16, 17). Therefore, effective and individualized ICI treatments are urgently needed for AML patients. T cell immunodeficiency is a common characteristic of AML, thus adoptive T cell immunotherapy has recently emerged as an effective and prospective strategy for improving anti-leukemia therapy (18). It is known that γδ T cells are a numerically small subset of T cells in human peripheral blood (PB), and upon activation, they perform the characteristic functions of both innate and adaptive immunity (19). Human γδ T cells have the ability to recognize a wide range of antigens in the absence of major histocompatibility complex molecules and can directly attack stressed cells *via* their cytotoxic activity or by indirectly enhancing the biological functions of other immune cells (20, 21). A growing body of evidence has demonstrated that γδ T cells could lead to cytotoxic activation in many types of solid tumors, including lymphoma, breast, prostate, and colon cancer (22, 23). Additionally, γδ T cells are essential for anti-leukemia function and have been proposed to have therapeutic potential for leukemia treatment (24, 25). However, not all of the γδ T cell subsets perform anti-leukemia functions. In contrast, some expanded γδ T cell clones and subsets may be related to poor outcome for leukemia (26). In addition, γδ T cells have different functional subsets, including regulatory T cell subsets that express the transcription factor forkhead box p3 (Foxp3) (27). Foxp3-positive αβ T cells are traditional Tregs, and these cells have been observed to possess an immune regulatory function in patients (28, 29). The regulatory subset of γδ T cells that express Foxp3, termed γδ regulatory T cells (γδ Tregs), has been reported to be at a low expression frequency in tumor-infiltrating leukocytes and human PB; however, the relevant underlying regulatory mechanism remains unclear.

It has been reported that Treg proliferation and its suppressive functions are regulated by the PD-1/PD-L1 pathway *via* a potentially novel mechanism (30). As it is known, PD-1 is an immunoreceptor expressed on activated T cells that negatively regulates antigen receptor signaling and mediates T cell suppression and dysfunction in leukemia (31). The PD-1/PD-L1 pathway plays a critical role in the prevention of abnormal autoimmune responses, and blockade of the PD-1/PD-L1 pathway was demonstrated to be an effective treatment for hematologic malignancies by clinical enhancement of the immune response (32, 33). Furthermore, the PD-1/PD-L1 pathway maintains Foxp3 stability by inhibiting degradation *via* downregulation of the endo-lysosomal protease asparaginyl endopeptidase (34). Our previous study reported that higher expression of ICIs in bone marrow (BM) leukemia cells in AML patients correlates with poor outcome (35, 36). Moreover, higher PD-1 expression was detected on exhausted CD8^+^ T cells in AML patients (37). However, whether PD-1 expression correlates with γδ Tregs and influences prognosis in AML needs to be further studied. In this study, we characterized increased PD-1^+^Foxp3^+^ γδ T cells in patients with *de novo* AML, which may be relevant to poor clinical outcome.

## Materials and methods

### Acquisition of the TCGA dataset

From the cancer genome atlas (TCGA; https://cancergenome.nih.gov/) database, level 3 RNA-seq data from 167 AML patients was downloaded using UCSC XENA (https://xenabrowser.net/datapages/) related data analysis (34). RNA-seq data are presented in the form of log_2_ (RPKM+1). Subsequently, RNA-seq data were assigned to a training group for analysis. Because the TCGA dataset is publicly available, no local ethics committee approval was required (**Supplementary Table 1**).

### Samples

PB samples were collected with consent from 36 *de novo* AML patients, including 17 males and 19 females with a median age of 53.5 years (range: 18-86 years). PB from 25 age-matched healthy individuals (HIs) who had no acute or chronic infectious diseases, autoimmune diseases, or tumors, including 13 males and 12 females with a median age of 55 years (range: 25-70 years), were recruited as healthy controls. There are 21 PB samples were detected by flow cytometry and the remain 15 PB samples were used as the training cohort by quantitative real-time PCR (qRT-PCR). The overall survival was defined as the time from the date of diagnosis to the date of death or last follow- up time. The clinical information of AML patients was listed in **Supplementary Table 2**. All procedures were conducted according to the guidelines of the Medical Ethics committees of the health bureau of Guangdong Province in China, and ethical approval was obtained from the Ethics Committee of the First Affiliated Hospital of Jinan University.

### Flow cytometry

Peripheral blood mononuclear cells (PBMCs) were isolated from AML patients and HIs and then incubated with the following antibodies: CD45-V450 (clone H130), CD3-Alexa Fluor^®^ 700 (clone SP34-2), TCR γδ-PE-Cy7 (clone B1), Vδ1-FITC (clone TS8.2), Vδ2-PerCP (clone B6), PD-1-PE (clone EH12.2H7), Foxp3-Alexa Fluor^®^ 647 (clone 150D), PE isotype control (clone MOPC-21), and Alexa Fluor^®^ 647 isotype control (clone 259D/C7) (BioLegend, SanDiego, USA; BD Biosciences, San Jose, USA; Abcam Cambridge, UK). First, cells obtained from fresh PB samples were stained with surface markers including CD45, CD3, TCR γδ, Vδ1, Vδ2, and PD-1 at 4°C in the dark for 30 minutes. For Foxp3 expression detection, anti-Foxp3 antibody and Foxp3 Fix/Perm buffer were used according to the manufacturer’s instructions. The gating standards for PD-1 and Foxp3 were set using the isotype controls recommended by the manufacturer. A total of 30,000-50,000 CD3^+^ cells were collected with a BD FACS VERSE flow cytometer (BD Biosciences, San Jose, USA), and data were analyzed by Flowjo software (Flowjo LLC, USA) (38).

### γδ T cell sorting and quantitative real-time PCR (qRT-PCR)

γδ T cells were sorted from PBMCs using a γδ T cell monoclonal antibody and the MACS magnetic cell sorting technique (Miltenyi Biotec, Bergisch Gladbach, Germany). RNA was reverse transcribed into first-strand cDNA with random hexamer primers. qRT-PCR using the SYBR Green I technique was used to examine the *FOXP3* and *PD-1* gene expression level in cDNA from γδ T cells with the β_2_ microglobulin gene serving as an endogenous reference. The primers were purchased from Invitrogen Biotechnology Co. Ltd. (Shanghai, China) (**Supplementary Table 3**). The relative mRNA expression level was calculated using the 2^-△Ct^× 100% method (39).

### Statistical analysis

All data are represented as medians. Comparisons between the different γδ T cell populations and differences in mRNA expression between two groups were analyzed by the Mann Whitney *U* test for non-parametric values. Spearman rank correlations and linear regression analyses were used to estimate the correlation between quantitative parameters. Mann Whitney *U* test, Spearman rank correlations, and linear regression analyses were performed by the R package “ggplot2”. Cox regression analysis was used to explore associations between the frequencies of γδ T cells and their subsets and the outcome of patients with *de novo* AML. The explanatory variables included the proportions of the Foxp3^+^, PD-1^+^, and PD-1^+^Foxp3^+^ subsets in γδ T cells. Odds ratios and 95% confidence intervals were also calculated. Statistical analyses were performed with SPSS 13.0 statistical software and R package “ggplot2”. *P* < 0.05 was considered significant.

## Results

### Co-expression characteristics of the *FOXP3* and *PD-1* genes in AML

**Figure 1:**
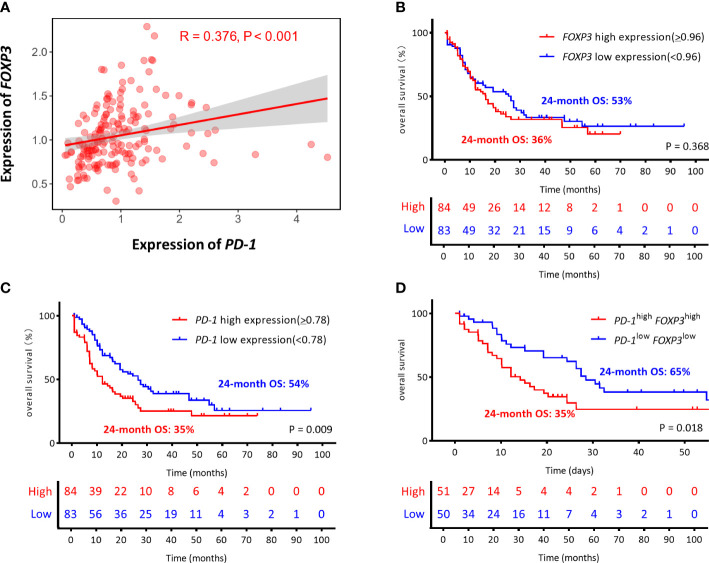
The correlation and the prognostic between the expression of *PD-1* and *FOXP3* genes in AML from the TCGA (The Cancer Genome Atlas). **(A)** Correlation of the expression levels of *PD-1* and *FOXP3*. **(B–D)** The optimal cutoff values were based on the median gene expression levels, the *FOXP3* and *PD-1* genes were divided into high expression (red line) and low expression (blue line) groups, which were plotted in Kaplan-Meier curves (top) with the number at risk AML patients (bottom). Kaplan-Meier curves are shown for single *FOXP3* high or low expression, single *PD-1* high or low expression, and co-high or co-low expression of *PD-1/FOXP3*. **(B)** *FOXP3* ^high^ vs. *FOXP3* ^low^, 24-month OS: 36% vs. 53% *P* = 0.368. **(C)** *PD-1* ^high^ vs. *PD-1* ^low^, 24-month OS: 35% vs. 54% *P* = 0.009. **(D)** *PD-1* ^high^ *FOXP3* ^high^ vs. *PD-1* ^low^ *FOXP3* ^low^, 24-month OS: 35% vs. 65% *P* = 0.018.

Previous studies from our research group have shown that higher PD-1 expression is related to poor OS for AML patients (34). In this study, we first explored the correlation between the expression and prognostic value of *PD-1* and *FOXP3* for AML patients by analyzing RNA-seq data from 167 AML patients in the TCGA database. According to the media expression levels of *FOXP3/PD-1* genes, AML patients were divided into high-, low-, co-high, and co-low expression groups to plot and compare Kaplan-Meier curves. Our initial results demonstrated there was a positive correlation between *PD-1* and *FOXP3* (R = 0.376, *P*<0.001) (**Figure 1A**). Subsequently, we found no significant correlation between the *FOXP3* gene expression level and OS (*FOXP3* ^high^ vs. *FOXP3* ^low^, 24-month OS: 36% vs 53%, *P* = 0.368) (**Figure 1B**), while a significant association between the *PD-1* gene and OS was found (*PD-1* ^high^ vs. *PD-1* ^low^, 24-month OS: 35% vs 54%, *P* = 0.009) (**Figure 1C**). More importantly, AML patients with higher *FOXP3* and *PD-1* co-expression had worse OS (*PD-1* ^high^ *FOXP3* ^high^ vs. *PD-1* ^low^ *FOXP3* ^low^, 24-month OS: 35% vs 65%, *P* = 0.018) (**Figure 1D**).

### Skewed distribution of γδ T cells and increased Foxp3^+^/PD-1^+^ γδ T cells in AML patients

**Figure 2:**
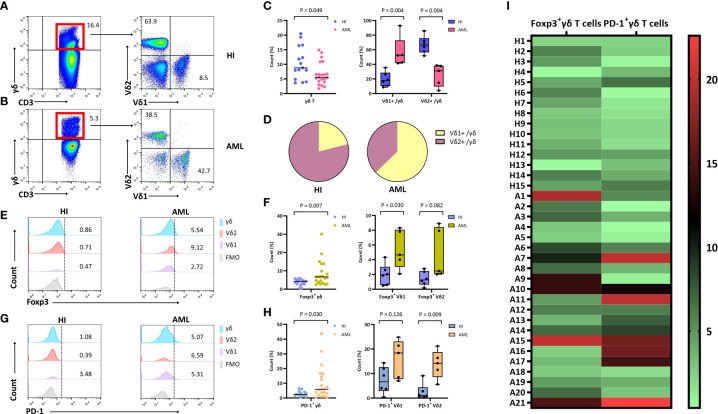
Distribution and frequency of Foxp3 and PD-1 expression in γδ, Vδ1, and Vδ2 cell subsets in PB from patients with *de novo* AML. **(A, B)** γδ T cells were gated by CD3^+^ T cells, further identifying Vδ1 and Vδ2 cell subsets from a healthy donor and a patient with *de novo* AML by flow cytometry analysis. **(C)** Comparison of the percentage of γδ, Vδ1, and Vδ2 cell subsets in AML patients compared with HIs. **(D)** The pie chart representing the distribution of Vδ1 and Vδ2 cells in AML patients and healthy individuals (HIs). **(E)** Detection of Foxp3 expression in γδ, Vδ1, and Vδ2 cell subsets from a healthy donor and an AML patient. **(F)** Comparison of the percentage of Foxp3^+^ γδ, Vδ1, and Vδ2 cell subsets in AML patients compared with HIs. **(G)** Detection of PD-1 expression in γδ, Vδ1, and Vδ2 cell subsets from a healthy donor and an AML patient. **(H)** Comparison of the percentage of PD-1^+^ γδ, Vδ1, and Vδ2 cell subsets in AML patients compared with HIs. **(I)** Heatmap representing the frequency of the Foxp3^+^ and PD-1^+^ γδ T subsets in patients with AML (A1-A21) compared with HIs (H1-H15).

To compare the frequency of γδ T cells and their subsets in AML patients with that in HIs, seven different antibodies were used for detection. CD45^high^ lymphocytes expressing CD3 and γδ TCRs were gated as CD3^+^ and γδ T cells, respectively. The γδ T cells were then divided into two subsets based on the expression of Vδ1 and Vδ2. A decreased trend for the total γδ T cell proportion in CD3^+^ T cells from 21 patients with *de novo* AML when compared with 15 HIs (**Figures 2A, B**) (median: 5.5% *vs.* 8.8%, *P* = 0.049) was found together with an increased proportion of the Vδ1 subgroup (median: 52.3% vs 17.7%, *P* = 0.004) and a decreased proportion of the Vδ2 T cell subgroup in the total γδ T cell population (median: 31.0% *vs.* 66.3%, *P* = 0.004) (**Figures 2C, D**). These results indicate a significantly decreased proportion of total γδ T cells with a higher frequency of the Vδ1 subset, and there was a lower trend for the Vδ2 subset in AML patients. Next, we examined the expression of Foxp3 in the γδ T cell subsets. A significantly higher percentage of Foxp3^+^ γδ T cells in the CD3^+^ T cell population (median: 6.7% *vs.* 4.2%, *P* = 0.007) and Foxp3^+^ Vδ1 in the γδ T cell population (median: 4.7% *vs.* 2.0%, *P* = 0.030) was found in AML patients. A high tendency for Foxp3^+^ Vδ2 in γδ T cells was also found; however, this did not appear to be statistically significant (median: 2.5% *vs.* 1.3%, *P* = 0.082) (**Figures 2E, F**). We further compared the PD-1 distribution in different γδ T cell subsets. It was noted that there was a dramatically increased trend in total PD-1^+^ γδ T cells in the CD3^+^ T cell population (median: 5.8% *vs.*2.4%), PD-1^+^ Vδ1 cells in the γδ T cell population (median: 18.4% *vs.* 6.6%), and PD-1^+^ Vδ2 cells in the γδ T cells population (median: 14.2% vs. 1.0%) (*P* = 0.030*, P* = 0.126, and *P* = 0.009, respectively) (**Figures 2G–I**).

### A high proportion of PD-1 on Foxp3^+^ γδ T cells in AML patients

**Figure 3:**
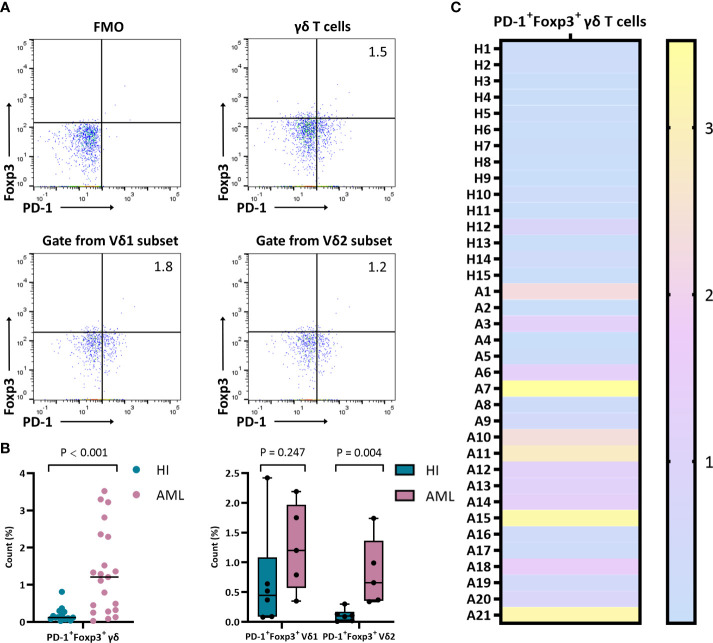
Distribution and frequency of co-expression with PD-1 and Foxp3 in γδ, Vδ1, and Vδ2 cell subsets. **(A)** Detection of co-expression with PD-1 and Foxp3 in γδ, Vδ1, and Vδ2 cell subsets from a patient with *de novo* AML by flow cytometry analysis. **(B)** Comparison of the percentage of PD-1^+^Foxp3^+^ in γδ, Vδ1, and Vδ2 cell subsets from *de novo* AML patients compared with HIs. **(C)** Heatmap representing the frequency of the PD-1^+^Foxp3^+^ γδ T subset in HIs (H1-H15) and patients with *de novo* AML (A1-A21).

To better understand PD-1 expression on Foxp3^+^ γδ T cells, we further examined and compared the frequency of PD-1 on this subset. We detected the percentage of PD-1^+^Foxp3^+^ γδ T cells in different samples by flow cytometry. Compared with HIs, the PD-1 proportion in the Foxp3^+^ γδ T cell population was significantly increased (median: 1.2% *vs.* 0.1%, *P* < 0.001), and the Foxp3^+^ Vδ2 subset was also increased in AML patients (median: 0.7% *vs.* 0.1%, *P* = 0.004) (**Figures 3A, B**). However, there was no significant difference in the Foxp3^+^ Vδ1 subset (median: 1.2% *vs.* 0.4%, *P* = 0.247) between AML patients and HIs (**Figures 3B, C**).

**Figure 4:**
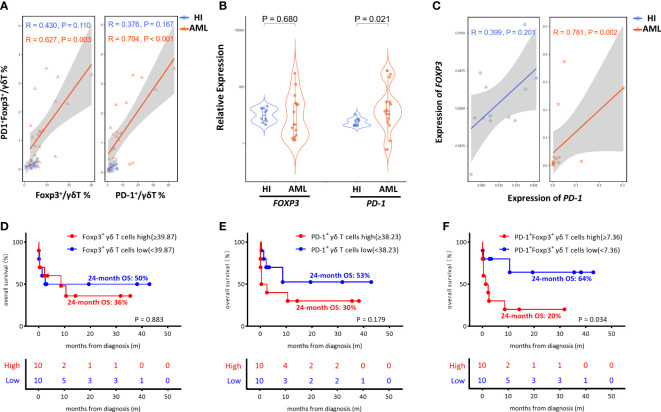
Correlation and OS analysis of PD-1 and Foxp3 in γδ T cells from *de novo* AML patients. **(A)** Correlation of the frequency of the Foxp3^+^, PD-1^+^, and PD-1^+^ Foxp3^+^ γδ T subsets between *de novo* AML patients and HIs. **(B)** The expression levels of the *FOXP3* and *PD-1* genes in γδ T cells from *de novo* AML patients compared to HIs. **(C)** Correlation analysis of the *FOXP3* and *PD-1* genes. **(D–F)** Correlation of the OS of *de novo* AML patients stratified by high and low Foxp3^+^ γδ T subsets, high and low PD-1^+^ γδ T subsets and high and low PD-1^+^ Foxp3^+^ γδ T subsets. **(D)** Foxp3^+ high^ vs. Foxp3^+ low^, 24-month OS: 36% vs. 50% *P* = 0.883. **(E)** PD-1^+high^ vs. PD-1^+low^, 24-month OS: 30% vs. 53% *P* = 0.179. **(F)** PD-1^+^ Foxp3^+high^ vs. PD-1^+^ Foxp3^+low^, 24-month OS: 20% vs. 64% *P* = 0.034.

Notably, we found a prominent positive correlation between PD-1^+^Foxp3^+^ γδ T cells and the Foxp3^+^ γδ T cell proportion (*R* = 0.627, *P* = 0.003) in AML patients, and there was no significant correlation between the proportions of those two groups (R = 0.430, *P* = 0.110) in HIs. Moreover, a positive correlation between the PD-1^+^Foxp3^+^ γδ and PD-1^+^ γδ T cell proportion was also found in the AML patients (*R* = 0.704, *P* < 0.001), while there was no significant correlation in HIs (*R*= 0.376, *P* = 0.167) (**Figure 4A**). We also analyzed the expression levels of the *FOXP3* and *PD-1* genes in γδ T cells from *de novo* AML patients and compared these results with that of HIs. Remarkably, higher *PD-1* expression levels were detected in the *de novo* AML group compared with the HI group (*P* = 0.021), and a higher *FOXP3* expression trend was also found (*P* = 0.680) (**Figure 4B**). Moreover, a positive correlation between *FOXP3* and *PD-1* gene expression in γδ T cells (*R* = 0.781, *P* = 0.002) from *de novo* AML patients was found, but there was no significant correlation in HIs (*r* = 0.399, *P* = 0.201) (**Figure 4C**). Overall, these results indicated a significantly higher proportion of γδ Treg cells and a novel Foxp3^+^ Vδ2 subset expressing PD-1 in patients with AML.

### Relevance of PD-1^+^Foxp3^+^ γδ T cells in AML clinical outcome

Despite the increased insight into the phenotype of γδ T cells, whether this phenotype correlates with clinical outcome remains poorly understood. It is unclear whether the increase in the novel PD-1^+^Foxp3^+^ γδ T cell subset affects AML clinical outcome. Therefore, we assessed the clinical outcomes of the 21 AML patients including one patient who refused therapy and voluntarily left the hospital. We further analyzed the association among AML outcome, OS, and proportions of the Foxp3^+^ population, PD-1^+^ population, and PD-1^+^Foxp3^+^ population in the γδ T cell subset. We divided the patients into high and low groups based on the median frequency of the γδ T cell subset. There was no significant difference in the Foxp3^+^ γδ and PD-1^+^ γδ T cell groups (Foxp3^+high^ vs. Foxp3^+low^ 24-month OS: 36% vs. 50% *P* = 0.883; PD-1^+high^ vs. PD-1^+low^ 24-month OS: 30% vs. 53%, *P* = 0.179) (**Figures 4D, E**). Strikingly, AML patients with a high level of PD-1^+^Foxp3^+^ γδ T cells were found to have poor OS (PD-1^+^Foxp3^+high^ vs. PD-1^+^Foxp3^+low^, 24-month OS 20% vs. 64%, *P* = 0.034), suggesting that the high proportion of the PD-1^+^Foxp3^+^ γδ T subset was associated with poor clinical outcome (**Figure 4F**).

## Discussion

Although γδ T cells represent only a minor fraction of T cells in PB, the potent antitumor cytotoxic activity of these cells is crucial for establishing and initiating immune responses (40, 41). Several clinical trials involving γδ T cell-based immunotherapies have demonstrated promising effects in solid tumors and hematological malignancies (42, 43). However, the clinical benefits appear to be mild to moderate at best. γδ T cells might have dual effects, and researchers have turned their attention away from the well-known immune effector role of γδ T cells and toward the newfound immunosuppressive regulatory role. Increasing data have demonstrated the heterogeneity of γδ T cells; however, little is known about the distribution of γδ T cells and their subsets in AML patients. Our previous study found the altered expression pattern and clonality of the γδ T cell receptor repertoire and identified that some clonally expanded γδ T cell clones might be related to the immune response and clinical outcome of AML patients (44, 45). Our previous study described the prognostic value of immune checkpoint inhibitors for AML patients by analyzing RNA-seq from the TCGA database and further validated results indicated that high expression of PD-1, PD-L1, and PD-L2 in the BM leukemia cells of AML patients correlated with poor outcomes (34). To the best of our knowledge, we first explored the association between the expression levels of the *PD-1* and *FOXP3* genes and the OS in the BM leukemia cells AML patients based on the TCGA database and described the expression pattern correlated with the poor OS. Besides, our further study found that PD-1 on CD8^+^ T cells was generally expressed higher in PB and BM from *de novo* and relapse-refractory AML patients, while it was partially recovered in complete remission patients (36). These results may be due to a novel change in the expression patterns of ICIs in AML, which suggests that there are different roles of PD-1 on T cells and their subsets. However, the expression profiles and clinical prognosis correlation of PD-1 in γδ T cells and their subsets in AML patients have not been clearly defined.

Because PB was more beneficial to access to observation and evaluation, in this study, we comprehensively compared the proportions of γδ T cells from PB samples, and these data indicate a significantly decreased frequency of total γδ T cells with a higher proportion of the Vδ1^+^ subset, and there was a lower trend for the Vδ2^+^ subset in AML patients. It is well known that Vδ2 T cells play a critical role in anti-tumor effects, and the skewed distribution of γδ T cells with a low number of Vδ2^+^ T cells may be a reason for γδ T cell dysfunction in AML. This abnormal expression pattern was also reported to be associated with poor prognosis in chronic lymphocytic leukemia (46). Our results also demonstrate an increasing trend in the Foxp3^+^ T cell subsets in the Vδ1 and Vδ2 T cell populations, which might be related to the primary reason for leukemia immunosuppression. Brauneck et al. also reported that BM-infiltrating Vδ1 T cells showed an increased terminally differentiated cell population in AML in comparison to healthy donors with an aberrant subpopulation of CD27^−^CD45RA^+^ cells (47). Our previous study revealed that the co-inhibitory TIGIT axis may be involved in the regulation of Foxp3^+^ γδ Treg cells and indicate the clinical progression and prognosis of AML patients with different clinical statuses (48, 49). Thus, it would be worth further characterizing other inhibitory phenotypes of the γδ T cell subsets in newly diagnosed AML, which may be an advantage of receiving immunotherapy. Besides, it is important to compare the frequency of γδ T cells between PB and BM in AML patients in our further study.

Previously, we reported an increase in the PD-1 expression frequency in αβ^+^ T cells in AML patients, and the prognosis of these patients was significantly worse (50). However, the expression profiles and clinical prognosis correlation of PD-1 in γδ T cells and their subsets in AML patients have not been clearly defined. However, litter is known about the expression patterns of PD-1 and Foxp3 and whether there are immunosuppressive regulatory subgroups γδ T cells in patients with AML. Hence, we further sought to investigate the proportion of PD-1 on γδ Treg cells. A significantly higher proportion of γδ Treg cells and a novel Foxp3^+^ Vδ2 subset expressing PD-1 were observed. Recent research has also reported that Vδ2 γδ T cells exhibit the exhausted state *via* PD-1 upregulation at diagnosis in AML patients (51). Most importantly, the expression proportions of the PD1^+^ γδ T cell and γδ Treg subgroups were positively correlated with the PD1^+^ γδ Treg subgroup. Interestingly, the correlation and pattern of *PD-1* and *FOXP3* from the TCGA database were again confirmed and existed in the γδ T cells from AML PB samples. We found that a higher co-expression level of *PD-1* and *FOXP3* was associated with poor AML clinical outcomes. This result may be supported by the findings of Dyck et al. who found a positive correlation between high PD-1 expression and increased tumor-infiltrating Tregs, and blocking PD-1 could effectively enhance anti-tumor immunity (52). There were also similar results from Ahearne et al. who reported that infiltration by both the Foxp3^+^CD4^+^ and PD-1^+^CD4^+^ T cell subsets was correlated with the prognosis of patients with diffuse large B-cell lymphoma (53), and Suresh et al., who found that PD-1^+^ and Foxp3^+^ T cell reduction might correlate with survival and serve as a predictive biomarker for hepatocellular carcinoma patients undergoing sorafenib therapy (54). In addition, further studies should elucidate the complex mechanisms of the PD-1 axis in suppressing γδ T cell function in the tumor microenvironment.

Taken together, in addition to the previously reported increase in ICIs in αβ^+^ T cells and higher T cell exhaustion status in AML patients, we further speculate that the high frequency of the PD-1^+^Foxp3^+^ γδ T subset is associated with poor clinical outcomes, which could reinforce evidence of a link between PD-1 and Foxp3 in γδ T cells. These results support the idea that there is a diverse and functional heterogeneity of γδ T cells, and the combined application of PD-1 and Foxp3 in novel targeted therapies may improve AML patient survival. This finding may partially explain how γδ T cells may be polarized from anti-leukemia to protumor cells, which appears to be the likely reason for mild efficacy in response to leukemia cells.

## Conclusion

**Figure 5:**
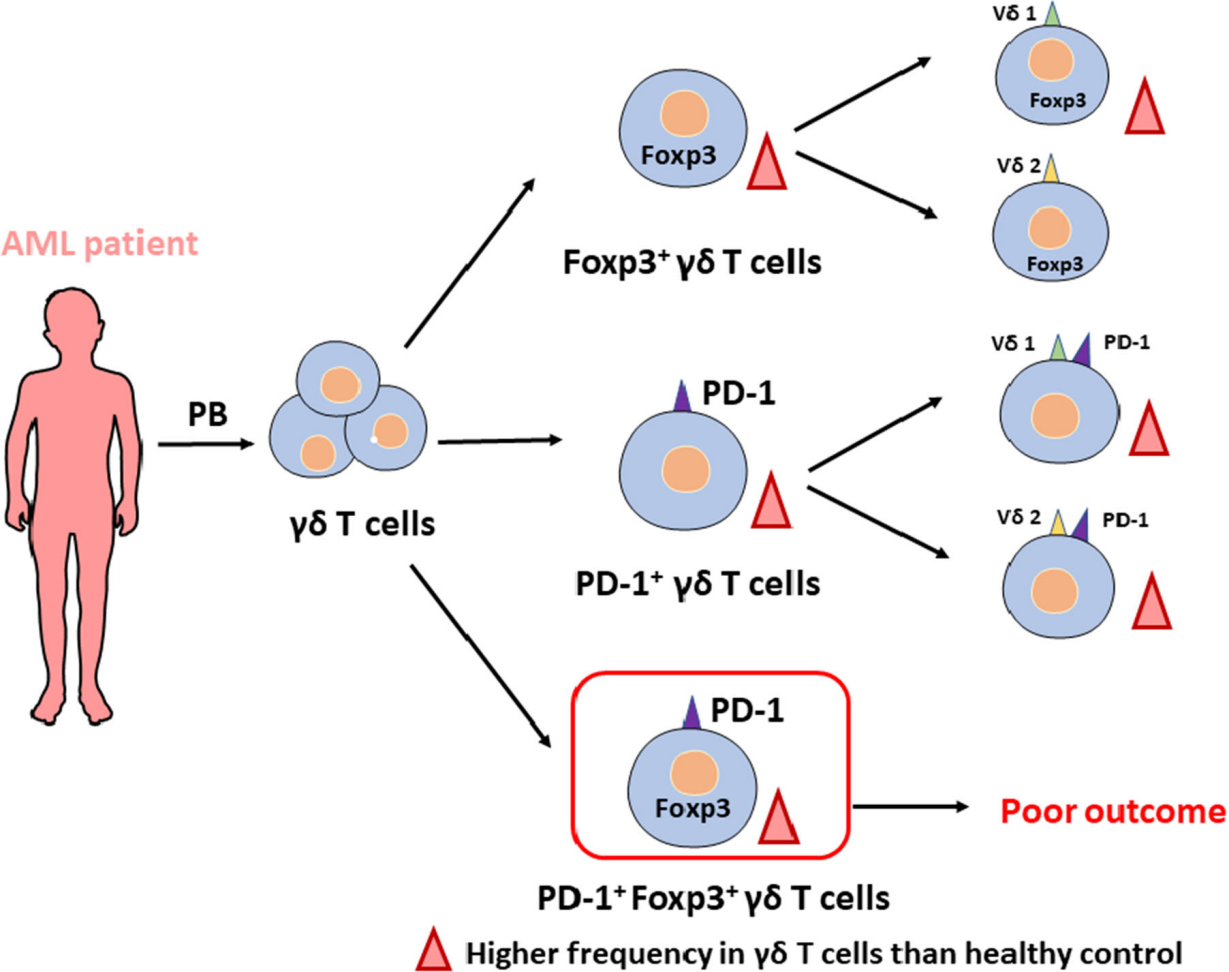
Overview of alterations in γδ T cell subsets in patients with AML.

We characterized the skewed distribution of γδ T cells with an inversion in the proportion of the Vδ1/Vδ2 T cell subset in AML patients, and we found a significant increase in the PD-1^+^Foxp3^+^ γδ T cell subset in AML, which was associated with poor clinical outcome (**Figure 5**). Our study facilitates a better understanding of the interaction of γδ T cells in AML patients and provides predictive value for the study of AML patients. However, more *in vivo* experiments are required to elucidate the function of the novel PD-1^+^Foxp3^+^ γδ T cell subset and investigate whether it could serve as a target for immunotherapy.

## Data availability statement

The original contributions presented in the study are included in the article/**Supplementary Material**. Further inquiries can be directed to the corresponding authors.

## Ethics statement

The studies involving human participants were reviewed and approved by Ethics Committee of School of Medicine of Jinan University. The patients/participants provided their written informed consent to participate in this study.

## Author contributions

YQL, XLW, and ZYJ contributed to the concept development and study design. DQ, YZ, HTZ, and XFW performed the laboratory studies. JC, JL, and XTL collected the clinical data. ZYJ, JMZ, DQ, XJ, and WBZ participated in the manuscript and figure preparation. YQL and XLW coordinated the study and helped draft the manuscript. All authors read and approved the final manuscript.

## References

[1] Kantarjian H Kadia T DiNardo C Daver N Borthakur G Jabbour E. Acute myeloid leukemia: Current progress and future directions. Blood Cancer J (2021) 11(2):41. doi: 10.1038/s41408-021-00425-3 33619261 PMC7900255

[2] Liu H. Emerging agents and regimens for aml. J Hematol Oncol (2021) 14(1):49. doi: 10.1186/s13045-021-01062-w 33757574 PMC7989091

[3] Döhner H Wei AH Appelbaum FR Craddock C DiNardo CD Dombret H. Diagnosis and management of aml in adults: 2022 recommendations from an international expert panel on behalf of the eln. Blood (2022) 140(12):1345–77. doi: 10.1182/blood.2022016867 35797463

[4] Khoury JD Solary E Abla O Akkari Y Alaggio R Apperley JF. The 5th edition of the world health organization classification of haematolymphoid tumours: Myeloid and Histiocytic/Dendritic neoplasms. Leukemia (2022) 36(7):1703–19. doi: 10.1038/s41375-022-01613-1 PMC9252913 35732831

[5] Hourigan CS Gale RP Gormley NJ Ossenkoppele GJ Walter RB. Measurable residual disease testing in acute myeloid leukaemia. Leukemia (2017) 31(7):1482–90. doi: 10.1038/leu.2017.113 28386105

[6] Yu J Li Y Zhang D Wan D Jiang Z. Clinical implications of recurrent gene mutations in acute myeloid leukemia. Exp Hematol Oncol (2020) 9(1):4. doi: 10.1186/s40164-020-00161-7 32231866 PMC7099827

[7] Stein EM DiNardo CD Pollyea DA Fathi AT Roboz GJ Altman JK. Enasidenib in mutant Idh2 relapsed or refractory acute myeloid leukemia. (2017) 130(6):722–31. doi: 10.1182/blood-2017-04-779405 PMC5572791 28588020

[8] DiNardo CD Stein EM de Botton S Roboz GJ Altman JK Mims AS. Durable remissions with ivosidenib in Idh1-mutated relapsed or refractory aml. (2018) 378(25):2386–98. doi: 10.1056/NEJMoa1716984 29860938

[9] Cortes JE Khaled S Martinelli G Perl AE Ganguly S Russell N. Quizartinib versus salvage chemotherapy in relapsed or refractory Flt3-itd acute myeloid leukaemia (Quantum-r): A multicentre, randomised, controlled, open-label, phase 3 trial. Lancet Oncol (2019) 20(7):984–97. doi: 10.1016/S1470-2045(19)30150-0 31175001

[10] Perl AE Martinelli G Cortes JE Neubauer A Berman E Paolini S. Gilteritinib or chemotherapy for relapsed or refractory Flt3-mutated aml. N Engl J Med (2019). 381(18):1728–40. doi: 10.1056/NEJMoa1902688 31665578

[11] Stein EM DiNardo CD Fathi AT Pollyea DA Stone RM Altman JK. Molecular remission and response patterns in patients with mutant-Idh2 acute myeloid leukemia treated with enasidenib. Blood (2019) 133(7):676–87. doi: 10.1182/blood-2018-08-869008 PMC6384189 30510081

[12] Duval M Klein JP He W Cahn JY Cairo M Camitta BM. Hematopoietic stem-cell transplantation for acute leukemia in relapse or primary induction failure. J Clin Oncol Off J Am Soc Clin Oncol (2010) 28(23):3730–8. doi: 10.1200/jco.2010.28.8852 PMC2917308 20625136

[13] Schlenk RF Döhner K Mack S Stoppel M Király F Götze K. Prospective evaluation of allogeneic hematopoietic stem-cell transplantation from matched related and matched unrelated donors in younger adults with high-risk acute myeloid leukemia: German-Austrian trial Amlhd98a. J Clin Oncol Off J Am Soc Clin Oncol (2010) 28(30):4642–8. doi: 10.1200/jco.2010.28.6856 20805454

[14] Liu D. Car-T "the living drugs", immune checkpoint inhibitors, and precision medicine: A new era of cancer therapy. J Hematol Oncol (2019) 12(1):113. doi: 10.1186/s13045-019-0819-1 31703740 PMC6842223

[15] Tan S Li D Zhu X. Cancer immunotherapy: Pros, cons and beyond. Biomedicine pharmacotherapy = Biomedecine pharmacotherapie (2020) 124:109821. doi: 10.1016/j.biopha.2020.109821 31962285

[16] Hansrivijit P Gale RP Barrett J Ciurea SO. Cellular therapy for acute myeloid leukemia - current status and future prospects. Blood Rev (2019) 37(1):100578. doi: 10.1016/j.blre.2019.05.002 31109711

[17] Salik B Smyth MJ Nakamura K. Targeting immune checkpoints in hematological malignancies. J Hematol Oncol (2020) 13(1):111. doi: 10.1186/s13045-020-00947-6 32787882 PMC7425174

[18] Liu H Pan C Song W Liu D Li Z Zheng L. Novel strategies for immuno-oncology breakthroughs with cell therapy. biomark Res (2021) 9(1):62. doi: 10.1186/s40364-021-00316-6 34332618 PMC8325826

[19] Vantourout P Hayday A. Six-of-the-Best: Unique contributions of Γδ T cells to immunology. Nat Rev Immunol (2013) 13(2):88–100. doi: 10.1038/nri3384 23348415 PMC3951794

[20] Scheper W Gründer C Kuball J. Multifunctional Γδ T cells and their receptors for targeted anticancer immunotherapy. Oncoimmunology (2013) 2(5):e23974. doi: 10.4161/onci.23974 23762790 PMC3667896

[21] Hayday AC. Γδ T cell update: Adaptate orchestrators of immune surveillance. J Immunol (Baltimore Md 1950) (2019) 203(2):311–20. doi: 10.4049/jimmunol.1800934 31285310

[22] Lafont V Sanchez F Laprevotte E Michaud HA Gros L Eliaou JF. Plasticity of Γδ T cells: Impact on the anti-tumor response. Front Immunol (2014) 5:622(1). doi: 10.3389/fimmu.2014.00622 25538706 PMC4259167

[23] Lo Presti E Di Mitri R Pizzolato G Mocciaro F Dieli F Meraviglia S. Γδ cells and tumor microenvironment: A helpful or a dangerous liason? J leukocyte Biol (2018) 103(3):485–92. doi: 10.1002/jlb.5mr0717-275rr 29345336

[24] Lo Presti E Pizzolato G Gulotta E Cocorullo G Gulotta G Dieli F. Current advances in Γδ T cell-based tumor immunotherapy. Front Immunol (2017) 8:1401(1). doi: 10.3389/fimmu.2017.01401 29163482 PMC5663908

[25] Siegers GM. Anti-leukemia activity of human gamma delta T cells. Oncoimmunology (2012) 1(2):237–9. doi: 10.4161/onci.1.2.18231 PMC3376996 22720255

[26] Jin Z Lan T Zhao Y Du J Chen J Lai J. Higher Tigit(+)Cd226(-) Γδ T cells in patients with acute myeloid leukemia. Immunol investigations (2022) 51(1):40–50. doi: 10.1080/08820139.2020.1806868 32819181

[27] Kang N Tang L Li X Wu D Li W Chen X. Identification and characterization of Foxp3(+) gammadelta T cells in mouse and human. Immunol Lett (2009) 125(2):105–13. doi: 10.1016/j.imlet.2009.06.005 19539651

[28] Li X Kang N Zhang X Dong X Wei W Cui L. Generation of human regulatory gammadelta T cells by tcrgammadelta stimulation in the presence of tgf-beta and their involvement in the pathogenesis of systemic lupus erythematosus. J Immunol (Baltimore Md 1950) (2011) 186(12):6693–700. doi: 10.4049/jimmunol.1002776 21562160

[29] Peng G Wang HY Peng W Kiniwa Y Seo KH Wang RF. Tumor-infiltrating gammadelta T cells suppress T and dendritic cell function *Via* mechanisms controlled by a unique toll-like receptor signaling pathway. Immunity (2007) 27(2):334–48. doi: 10.1016/j.immuni.2007.05.020 17656116

[30] Hu ZQ Zhao WH. Critical role of pd-1/Pd-L1 pathway in generation and function of follicular regulatory T cells. Cell Mol Immunol (2013) 10(4):286–8. doi: 10.1038/cmi.2013.15 PMC4003206 23624877

[31] Shi L Chen S Yang L Li Y. The role of pd-1 and pd-L1 in T-cell immune suppression in patients with hematological malignancies. J Hematol Oncol (2013) 6(1):74. doi: 10.1186/1756-8722-6-74 24283718 PMC3851976

[32] Marin-Acevedo JA Soyano AE Dholaria B Knutson KL Lou Y. Cancer immunotherapy beyond immune checkpoint inhibitors. J Hematol Oncol (2018) 11(1):8. doi: 10.1186/s13045-017-0552-6 29329556 PMC5767051

[33] Sun JY Zhang D Wu S Xu M Zhou X Lu XJ. Resistance to pd-1/Pd-L1 blockade cancer immunotherapy: Mechanisms, predictive factors, and future perspectives. biomark Res (2020) 8(1):35. doi: 10.1186/s40364-020-00212-5 32864132 PMC7450549

[34] Chen C Liang C Wang S Chio CL Zhang Y Zeng C. Expression patterns of immune checkpoints in acute myeloid leukemia. J Hematol Oncol (2020) 13(1):28. doi: 10.1186/s13045-020-00853-x 32245463 PMC7118887

[35] Liang C Zhao Y Chen C Huang S Deng T Zeng X. Higher tox genes expression is associated with poor overall survival for patients with acute myeloid leukemia. Front Oncol (2021) 11:740642(1). doi: 10.3389/fonc.2021.740642 34692519 PMC8532529

[36] Xu L Liu L Yao D Zeng X Zhang Y Lai J. Pd-1 and tigit are highly Co-expressed on Cd8(+) T cells in aml patient bone marrow. Front Oncol (2021) 11:686156(1). doi: 10.3389/fonc.2021.686156 34490086 PMC8416522

[37] Huang J Tan J Chen Y Huang S Xu L Zhang Y. A skewed distribution and increased pd-1+Vβ+Cd4+/Cd8+ T cells in patients with acute myeloid leukemia. J leukocyte Biol (2019) 106(3):725–32. doi: 10.1002/jlb.Ma0119-021r 31136687

[38] Tan J Chen S Huang J Chen Y Yang L Wang C. Increased exhausted Cd8(+) T cells with programmed death-1, T-cell immunoglobulin and mucin-Domain-Containing-3 phenotype in patients with multiple myeloma. Asia-Pacific J Clin Oncol (2018) 14(5):e266–e74. doi: 10.1111/ajco.13033 29943497

[39] Liao Z Lv X Liu S He Z Chen S Wang L. Different aberrant expression pattern of immune checkpoint receptors in patients with ptcl and Nk/T-cl. Asia-Pacific J Clin Oncol (2018) 14(5):e252–e8. doi: 10.1111/ajco.12850 29368793

[40] Van Acker HH Anguille S Van Tendeloo VF Lion E. Empowering gamma delta T cells with antitumor immunity by dendritic cell-based immunotherapy. Oncoimmunology (2015) 4(8):e1021538. doi: 10.1080/2162402x.2015.1021538 26405575 PMC4570126

[41] Van Acker HH Anguille S Willemen Y Van den Bergh JM Berneman ZN Lion E. Interleukin-15 enhances the proliferation, stimulatory phenotype, and antitumor effector functions of human gamma delta T cells. J Hematol Oncol (2016) 9(1):101. doi: 10.1186/s13045-016-0329-3 27686372 PMC5041439

[42] Fisher JP Heuijerjans J Yan M Gustafsson K Anderson J. Γδ T cells for cancer immunotherapy: A systematic review of clinical trials. Oncoimmunology (2014) 3(1):e27572. doi: 10.4161/onci.27572 24734216 PMC3984269

[43] Nada MH Wang H Workalemahu G Tanaka Y Morita CT. Enhancing adoptive cancer immunotherapy with Vγ2vδ2 T cells through pulse zoledronate stimulation. J immunotherapy Cancer (2017) 5(1):9. doi: 10.1186/s40425-017-0209-6 PMC5319075 28239463

[44] Jin Z Luo Q Lu S Wang X He Z Lai J. Oligoclonal expansion of tcr vδ T cells may be a potential immune biomarker for clinical outcome of acute myeloid leukemia. J Hematol Oncol (2016) 9(1):126. doi: 10.1186/s13045-016-0353-3 27863523 PMC5116135

[45] Kong X Zheng J Liu X Wang W Jiang X Chen J. High trgv 9 subfamily expression marks an improved overall survival in patients with acute myeloid leukemia. Front Immunol (2022) 13:823352(1). doi: 10.3389/fimmu.2022.823352 35222403 PMC8866455

[46] Coscia M Vitale C Peola S Foglietta M Rigoni M Griggio V. Dysfunctional Vγ9vδ2 T cells are negative prognosticators and markers of dysregulated mevalonate pathway activity in chronic lymphocytic leukemia cells. Blood (2012) 120(16):3271–9. doi: 10.1182/blood-2012-03-417519 22932792

[47] Brauneck F Weimer P Schulze Zur Wiesch J Weisel K Leypoldt L Vohwinkel G. Bone marrow-resident Vδ1 T cells Co-express tigit with pd-1, Tim-3 or Cd39 in aml and myeloma. Front Med (2021) 8:763773(1). doi: 10.3389/fmed.2021.763773 PMC8606547 34820398

[48] Jin Z Ye W Lan T Zhao Y Liu X Chen J. Characteristic of tigit and dnam-1 expression on Foxp3+ Γδ T cells in aml patients. BioMed Res Int (2020) 2020(1):4612952. doi: 10.1155/2020/4612952 32802845 PMC7403925

[49] Qiu D Liu X Wang W Jiang X Wu X Zheng J. Tigit axis: Novel immune checkpoints in anti-leukemia immunity. Clin Exp Med (2022) 1:1–10. doi: 10.1007/s10238-022-00817-0 35419661

[50] Tan J Yu Z Huang J Chen Y Huang S Yao D. Increased pd-1+Tim-3+ exhausted T cells in bone marrow may influence the clinical outcome of patients with aml. biomark Res (2020) 8(1):6. doi: 10.1186/s40364-020-0185-8 32082573 PMC7020501

[51] Tang L Wu J Li CG Jiang HW Xu M Du M. Characterization of immune dysfunction and identification of prognostic immune-related risk factors in acute myeloid leukemia. Clin Cancer Res an Off J Am Assoc Cancer Res (2020) 26(7):1763–72. doi: 10.1158/1078-0432.Ccr-19-3003 31911547

[52] Dyck L Wilk MM Raverdeau M Misiak A Boon L Mills KH. Anti-Pd-1 inhibits Foxp3(+) treg cell conversion and unleashes intratumoural effector T cells thereby enhancing the efficacy of a cancer vaccine in a mouse model. Cancer immunology immunotherapy CII (2016) 65(12):1491–8. doi: 10.1007/s00262-016-1906-6 PMC11028992 27680570

[53] Ahearne MJ Bhuller K Hew R Ibrahim H Naresh K Wagner SD. Expression of pd-1 (Cd279) and Foxp3 in diffuse Large b-cell lymphoma. Virchows Archiv an Int J Pathol (2014) 465(3):351–8. doi: 10.1007/s00428-014-1615-5 25011996

[54] Kalathil SG Lugade AA Miller A Iyer R Thanavala Y. Pd-1(+) and Foxp3(+) T cell reduction correlates with survival of hcc patients after sorafenib therapy. JCI Insight (2016) 1(11):e86182. doi: 10.1172/jci.insight.86182 27540594 PMC4986927

