# Supplementary material for: Increased PD-1+Foxp3+ γδ T cells associate with poor overall survival for patients with acute myeloid leukemia
Source: Front Oncol. 2022 Dec 15;12:1007565. doi: 10.3389/fonc.2022.1007565 (PMC9799959; doi:10.3389/fonc.2022.1007565)
Supplement: Supplementary file 1 [file DataSheet_1.pdf]

Supplementary Table 1 Clinical information for the AML patients.

Variables	Overall
Number	167
Age (median; range)	58 (18-88)
Gender (Male/Female)	88/78
FAB subtype (n = 167)	
M0	15
M1	36
M2	37
M3	16
M4	35
M5	21
M6	2
M7	3
Unclassified	2
Cytogenetic abnormality, n (%)	
No	84 (50.3)
Yes	67 (40.1)
Unknown	16 (9.6)
Risk Category, n (%)	
Favorable	33 (19.8)
Intermediate/Normal	97 (58.1)
Poor	35 (21.0)
Unknown	2 (1.2)
Status	
Alive	61
Dead	106

Supplementary Table 2 AML patient characteristics

Factor	AML
Number	36
Method	
Flow cytometry	21
qRT-PCR	15
Age (median; range)	53.5 (18-86)
Gender (Male/Female)	17/19
WBC ($\times 10^9$/L), (median; range)	21.7 (1.1-464.1)
BM blast cells (%), (median; range)	71.5 (27-91)
FAB subtype (n = 36)	
M0	/
M1	3
M2	12
M3	2
M4	7
M5	6
M6	/
M7	/
Undetermined	6
Gene mutation, n (%)	
FLT3	5 (13.9)
NPM1	2 (5.6)
PML/RARA	2 (5.6)
CEBPA	2 (5.6)
MLL	2 (5.6)
TP53	2 (5.6)
AML1/ETO	3 (8.3)
Others	4 (11.1)
No detected/Unknown	17 (47.2)
Cytogenetic abnormality, n (%)	
Normal	3 (8.3)
Abnormal	9 (25)
No detected/Unknown	24 (66.7)
Treatment	
Chemotherapy	21
HSCT	5
Supporting therapy	10
Status (method for flow cytometry)	
Alive	9
Dead	11
Loss to follow-up	1

Notes: AML: acute myeloid leukemia, WBC: white blood cell, RBC: red blood cell, PLT: platelet, BM blast cells: bone marrow blast cells, FAB: French-American-British, M0:

minimally differentiated AML, M1: AML without maturation, M2: AML with maturation, M3: acute promyelocytic leukemia, M4: acute myelomonocytic leukemia, M5: acute monocytic leukemia, M6: pure erythroid leukemia, M7: Acute megakaryoblastic leukemia, HSCT: hematopoietic stem cell transplantation, /: unknown.

Supplementary Table 3 qRT-PCR primers sequences

Primer name	Sequence
PD-1-forward	5'-CCAGGATGGTTCTTAGACTCCC-3'
PD-1-reverse	5'-TTTAGCACGAAGCTCTCCGAT-3'
FOXP3- forward	5'-CTGACCAAGGCTTCATCTGTG-3'
FOXP3- reverse	5'-ACTCTGGGAATGTGCTGTTTC-3'
β_2M- forward	5′-TACACTGAATTCACCCCCAC-3′
β_2M- reverse	5′-CATCCAATCCAAATGCGGCA-3′
